# Sparse-Observation Multi-Horizon Glaucoma Progression Forecasting with Biologically Constrained Temporal Consistency: A Glaucoma Case Study

**DOI:** 10.21203/rs.3.rs-10009600/v1

**Published:** 2026-06-25

**Authors:** Mousa Moradi, Jerry Cao-Xue, Asahi Fujita, Daniel L. Liebman, Alessandro Jammal, Mengyu Wang, Tobias Elze, Mohammad Eslami, Nazlee Zebardast

**Affiliations:** 1 Harvard Ophthalmology AI Lab, Schepens Eye Research Institute, Massachusetts Eye and Ear, Harvard Medical School, Boston, MA, United States; 2 Massachusetts Eye and Ear, Harvard Medical School, Boston, MA, United States

**Keywords:** Glaucoma progression, Temporal consistency, Multi-horizon, cpRNFL, VFTD

## Abstract

Glaucoma progression forecasting from sparse longitudinal data remains unsolved, as existing models require dense multi-year inputs and lack the biological constraints. We introduce a sparse-observation framework that predicts multi-horizon outcomes from only two visits, incorporating a novel TCMH (Temporally Consistent Multi-Horizon) loss that enforces monotonic risk ordering to reflect irreversible disease biology. Applied to glaucoma progression, we integrate circumpapillary retinal nerve fiber layer (cpRNFL) images, visual field total deviation (VFTD) maps, and clinical covariates through ConvNeXt architecture trained with TCMH loss. In 3,593 patients (13,087 sequences), our model achieved AUROC 0.968 and accuracy 0.947 for two-, three-, and four-year progression forecasting, with 10.2% better calibration than baseline and 0.0163 maximum demographic disparity. At 90% coverage, classification error remained 2.5%, enabling automated risk stratification with expert review for only uncertain cases. The model exceeded three independent specialist graders on specificity (0.98 vs. 0.57–0.73) on 108 held-out eyes. These results establish sparse-observation temporally consistent forecasting as a generalizable paradigm for calibrated long-horizon risk prediction in irreversible progressive diseases.

## Introduction

Artificial intelligence (AI) is transforming clinical medicine by enabling early detection and long-horizon forecasting of disease worsening from routinely collected patient data. Nowhere is this potential more consequential than in irreversible progressive diseases, where neuronal or retinal damage accumulates permanently and intervention opportunity narrows with each missed progressor visit^1–3^. Irreversible progressive eye diseases including glaucoma, age-related macular degeneration (AMD), and diabetic retinopathy collectively affect hundreds of millions of people worldwide^4–7^ as leading causes of permanent vision loss^8^, yet reliable AI-driven forecasting of disease worsening from sparse longitudinal observations remains an unsolved problem. Most existing models require dense multi-year visit sequences before generating trustworthy forecasts^9–11^, limiting applicability to patients without extended records and delaying intervention until substantial irreversible damage has already occurred.

The fundamental barrier is both clinical and methodological. Clinically, real-world longitudinal data present irregular visit intervals, missing modalities, and partially observed progression trajectories, conditions that most existing frameworks do not explicitly address^12,13^. Methodologically, multi-horizon predictions must respect the irreversible biology of progressive disease: risk estimates at later horizons should never fall below those at earlier horizons, yet standard binary cross-entropy losses treat each prediction horizon independently and provide no mechanism to enforce this constraint, particularly under sparse observation where the longitudinal trajectory signal is limited. As a result, models trained with conventional objectives can produce temporally inconsistent risk profiles that undermine clinical interpretability and downstream triage decisions.

Deep learning has enabled high-performing ophthalmic image analysis^7,14,15^ and longitudinal modeling approaches have shown promise for capturing disease trajectories^11,16^. Multimodal frameworks integrating structural and functional data have been explored for progression prediction^17,18^, yet critical gaps remain across prior works. Yousefi et al.^19^ used tabular circumpapillary retinal nerve fiber layer (cpRNFL) and visual field (VF) features with classical classifiers, achieving AUC 88% within two years but without imaging input and requiring multiple visits. Dixit et al.^9^ applied convolutional LSTMs to longitudinal VF sequences from over 11,000 eyes (accuracy 91–93%) excluding structural imaging. Tarcoveanu et al.^20^ reported approximately 92% accuracy on only approximately 150 eyes with limited generalizability. Afolabi et al.^21^ introduced an equity-aware EfficientNet on OCT B-scans without VF data, achieving only AUC 74%. Chen et al.^10^ demonstrated strong VF-Transformer performance without structural imaging fusion. Across all prior works, a critical underappreciated barrier persists: most longitudinal frameworks require three or more sequential examinations spanning multiple years as model input, and none jointly address sparse observation, temporal consistency across prediction horizons, calibrated uncertainty, and demographic equity within a single unified framework.

**Fig. 1.**
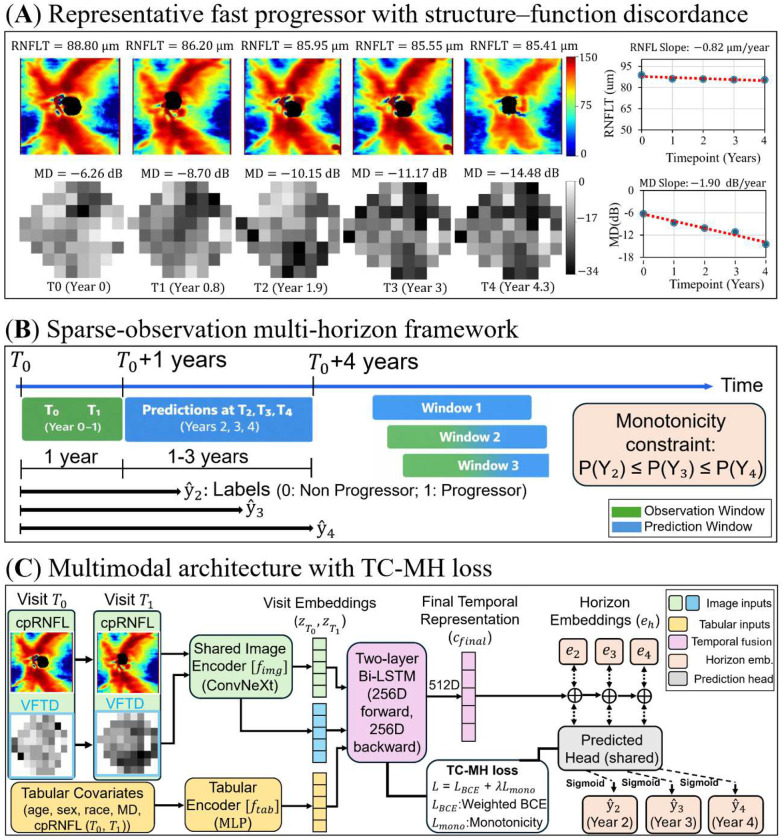
Sparse-observation multi-horizon glaucoma progression forecasting framework. (A) Representative fast progressor with structure–function discordance: cpRNFL thickness maps remain relatively stable over 4.3 years (RNFLT slope −0.82 μm/year), whereas VFTD maps show marked functional worsening (MD slope −1.90 dB/year), motivating forecasting from sparse observations. (B) Two visits, T0 and T1, within a 1-year observation window are used to predict progression at years 2, 3, and 4 using sliding prediction windows. The temporal consistency constraint P(Y2) ≤ P(Y3) ≤ P(Y4) reflects the irreversible nature of glaucoma progression. (C) Shared-weight ConvNeXt encodes cpRNFL and VFTD images, and an MLP encodes tabular covariates. Per-visit embeddings are fused by a two-layer bidirectional LSTM and combined with horizon embeddings to generate three sigmoid outputs. Training uses binary cross-entropy with a monotonicity penalty and patient-level five-fold cross-validation.

To address these gaps, we introduce a biologically constrained sparse-observation forecasting paradigm for longitudinal clinical AI, in which only two visits are used to estimate temporally ordered progression risk across two-, three-, and four-year horizons. We propose the Temporally Consistent Multi-Horizon (TCMH) loss, which combines multi-horizon supervision with a monotonicity regularization term that directly penalizes violations of P(Y_2_) ≤ P(Y_3_) ≤ P(Y_4_) across prediction horizons (Fig. 1B and 1C). Asymmetric temporal tolerances accommodate irregular clinical follow-up while preserving temporally ordered multi-horizon supervision. Horizon-conditioned output heads further condition each prediction on its temporal distance from the observation window, while entropy uncertainty quantification and risk-coverage analysis support reliability-aware clinical deployment. The proposed TCMH framework is benchmarked against four image backbones widely used in medical imaging (ConvNeXt^22^, Vision Transformer (ViT)^23^, MobileNet^24^, and EfficientNet^25^) within a shared Bi-LSTM temporal fusion framework across 3,593 patients (5,645 eyes; 13,087 sequences).

Glaucoma serves as the primary case study for evaluating TCMH, selected for three complementary reasons: its global burden exceeding 70 million affected individuals with projections to surpass 110 million by 2040^4^, the strictly irreversible and monotonically progressive nature of retinal ganglion cell loss that makes temporal consistency constraints biologically mandatory, and the availability of paired structural-functional longitudinal imaging, cpRNFL OCT and VF total deviation (VFTD) maps, that makes it an ideal testbed for sparse multimodal forecasting. Progressive cpRNFL thinning and VF deterioration on perimetry^1,26^ manifest across clinically distinct subtypes including primary open-angle (OAG), angle-closure (ACG), pseudoexfoliation (XFG), and pigmentary (PDG) glaucoma, each with different progression trajectories^27,28^, further challenging conventional progression labeling. Current practice relies on regression-based slope analyses of cpRNFL thickness (cpRNFLT) and VF mean deviation (MD) or TD, which are noisy, require years of follow-up to reach statistical significance^12,29^, and are sensitive to irregular visits and patient attrition^13^. Fig. 1A illustrates this challenge: a representative patient appeared stable at baseline (cpRNFL 88.80 μm, MD −6.26 dB) yet developed rapid functional decline (MD slope −1.90 dB/year over 4.3 years) despite structurally stable cpRNFL, underscoring the urgency of sparse-observation multimodal forecasting. Although glaucoma serves as our case study here, the TCMH framework is disease-agnostic: any condition characterized by irreversible biological progression, multimodal longitudinal monitoring, and irregular clinical follow-up is a candidate for this paradigm. Progressive conditions including AMD, diabetic retinopathy, multiple sclerosis, and Alzheimer's disease share these properties and represent natural targets for future extension.

Our contributions are as follows:

1. We introduce a sparse-observation forecasting paradigm that predicts 2-, 3-, and 4-year glaucoma progression outcomes from only two visits, substantially reducing the longitudinal data burden relative to prior multi-visit approaches.
2. We propose TCMH, a biologically constrained multi-horizon loss that enforces monotonic risk ordering across future horizons, aligning model predictions with the irreversible nature of glaucomatous neurodegeneration.
3. We demonstrate that TCMH improves not only discrimination but also calibration, uncertainty stratification, demographic consistency, and selective prediction, supporting reliability-aware clinical deployment from limited longitudinal data.
4. Through modality ablation and subtype analysis, we show that multimodal fusion provides disease-dependent benefit, with the greatest added value in angle-closure and pigmentary glaucoma.
5. We benchmark model predictions against three independent specialist graders on 108 held-out eyes, enabling direct quantification of human-AI concordance, inter-rater reliability, and confidence-aware triage under realistic clinical conditions.

More broadly, TCMH provides a general and transferable strategy for embedding irreversible disease biology into sparse longitudinal forecasting, with principles directly applicable wherever temporally ordered multi-horizon risk prediction is needed from minimal clinical observations.

## Results

### Patient Demographics and Clinical Characteristics

Table 1 illustrates baseline characteristics between progressors (N= 971 eyes) and non-progressors (N= 4,674 eyes). Progressors were significantly older (70.5±12.8 vs. 65.6±14.7 years), had similar gender distribution (51.1% vs. 58% female) but different racial composition, with White patients representing the majority in both groups (69.6% vs. 63.5%, *P* value< 0.001). Progressors demonstrated worse baseline disease with lower MD (−8.11±6.24 vs. −5.9±5.25 dB), thinner RNFL (59.4±9.5 vs. 77.9±11.3 μm), and faster deterioration rates for both MD slope (−0.013 vs. −0.005 dB/year) and cpRNFL slope (−0.530 vs. −0.264 μm/year) (all *P* values < 0.001).

### Performance Evaluation

Unless otherwise stated, all primary model-performance metrics are reported at the sequence level under 5-fold patient-level cross-validation (CV). We conducted five-fold cross-validation to evaluate model stability, establishing the robust predictive performance of the multimodal framework across the entire cohort of 5,645 eyes (971 progressors, 4,674 non-progressors). The cohort exhibited substantial class imbalance (progressor-to-non-progressor ratio approximately 1:4.8), addressed through weighted loss functions during training (see Method Section). Prediction uncertainty was quantified using Tsallis entropy with q = 0.25, selected for its superior sensitivity to low-probability deviations, making it particularly well-suited for imbalanced classification settings where minority-class predictions carry disproportionate clinical importance^30,31^ (see Methods Section).

Across all subgroups, ConvNeXt+TCMH achieved the strongest overall performance, with the highest discrimination (AUC-ROC 0.968, AUC-PR 0.938), accuracy (0.947), and F1-score (0.874), while maintaining the lowest prediction uncertainty (entropy 0.193). ConvNeXt performed second best, followed by ViT, whereas MobileNet and EfficientNet showed substantially lower discrimination and higher uncertainty (*P* value < 0.001). These results are shown in Supplementary Fig. S1.

**Fig. 3.**
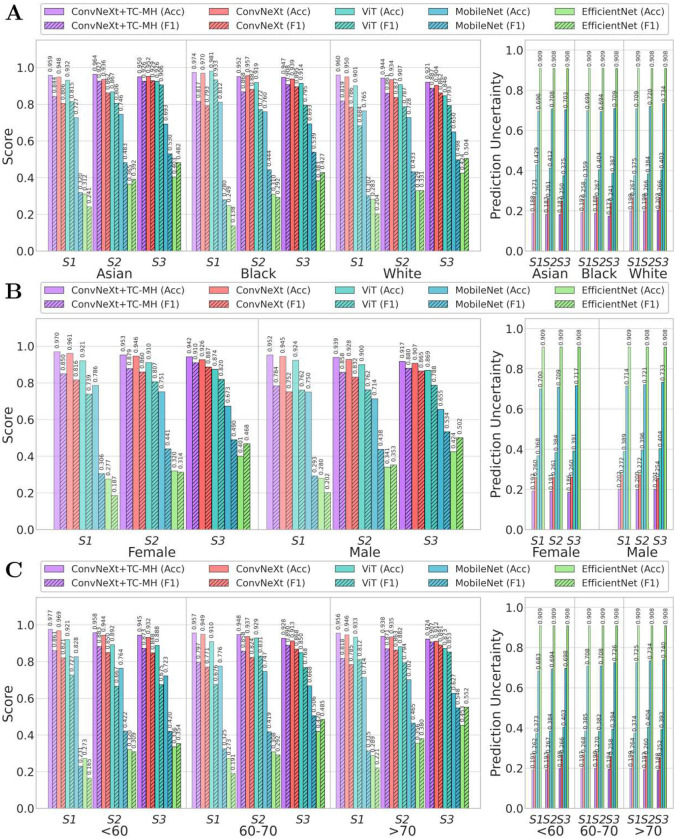
Severity-stratified subgroup performance and prediction uncertainty across all models. Accuracy (solid bars) and F1-score (hatched bars) are shown for ConvNeXt+TCMH, ConvNeXt, ViT, MobileNet, and EfficientNet across early (S1), moderate (S2), and severe (S3) disease stages, stratified by (A) race (Asian, Black, White), (B) sex (female, male), and (C) age group (<60, 60–70, >70 years). Right panels show mean Tsallis entropy uncertainty per subgroup and severity stage. ConvNeXt+TCMH consistently achieves the highest accuracy and F1-score with the lowest prediction uncertainty across all subgroups and severity stages, followed by ConvNeXt and ViT, while MobileNet and EfficientNet show substantially lower performance and higher uncertainty.

Severity-stratified subgroup analyses are summarized in Table 2 and detailed by demographic axis in Fig. 3 and Supplementary Fig. S2. Across severity stages, ConvNeXt+TCMH achieved the highest averaged AUROC, AUPRC, accuracy, and F1-score with the lowest uncertainty, with AUROC increasing from 0.950 at Stage 1 to 0.965 at Stage 3 and uncertainty remaining stable from 0.195 to 0.191.

Race-stratified analysis across disease severity (Fig. 3A; Supplementary Fig. S2A) revealed systematic performance advantages for ConvNeXt+TCMH and ConvNeXt, with gains amplifying at advanced disease stages. In Asian patients, ConvNeXt+TCMH showed near-equivalent early-stage performance to White patients (S1 accuracy 0.959 vs. 0.960) but progressive gains at moderate and severe stages (S2: accuracy 0.964, F1 0.926; S3: accuracy 0.950, F1 0.926). Black patients showed the strongest relative gains at S3 (accuracy 0.947, F1 0.908; +2.6% and +2.1% over White patients), consistent with greater model sensitivity to structural-functional discordance at advanced disease. MobileNet (accuracy 0.650–0.812) and EfficientNet (0.249–0.424) underperformed substantially across all subgroups and severity stages. Prediction uncertainty followed a clear hierarchy, with ConvNeXt+TCMH maintaining the lowest entropy (0.173–0.202) across all racial subgroups and severity stages. Aggregated across severity, ConvNeXt+TCMH achieved accuracy/F1 of 0.958/0.913, 0.958/0.883, and 0.943/0.865 in Asian, Black, and White patients respectively, with uncertainty consistently below all baselines (Supplementary Fig. S2A).

Gender-stratified analysis across disease severity (Fig. 3B; Supplementary Fig. S2B) revealed consistent performance advantages in female patients across all models and severity stages. ConvNeXt+TCMH demonstrated the most pronounced female advantage at early disease (S1 accuracy 0.970 vs. 0.952, F1 0.850 vs. 0.784; +1.8% and +6.6%), sustained through moderate and severe stages (S3: accuracy 0.942, F1 0.910; +2.5% and +3.0% over male patients). The ConvNeXt baseline followed a parallel pattern with uniformly lower absolute performance. MobileNet showed notably poor discriminative capacity in male patients at early disease (S1 F1 0.293), while EfficientNet performed weakest across all strata (accuracy 0.277–0.424). Prediction uncertainty remained low and stable for ConvNeXt+TCMH (0.186–0.202) and ConvNeXt (0.254–0.272) across both sexes. Aggregated across severity, ConvNeXt+TCMH achieved accuracy/F1 of 0.956/0.888 in female and 0.937/0.857 in male patients, with the lowest uncertainty across all models (0.190 and 0.201 respectively; Supplementary Fig. S2B).

Age-stratified analysis across disease severity (Fig. 3C; Supplementary Fig. S2C) revealed a nuanced pattern in which younger patients achieved higher accuracy, but older patients demonstrated superior F1-scores at advanced disease stages. In patients under 60, ConvNeXt+TCMH showed consistent accuracy advantages over those above 70 across all severity stages (+2.1% at S1, S2, and S3), yet older patients achieved higher F1 at severe disease (S3: 0.872 vs. 0.907, −3.5%), suggesting more reliable progressor identification where structural and functional biomarkers are unambiguous. This age-dependent F1 reversal was replicated in the ConvNeXt baseline, indicating a property of the clinical data distribution rather than a model-specific limitation. Prediction uncertainty remained remarkably stable for ConvNeXt+TCMH (0.188–0.199) and ConvNeXt (0.252–0.270) across all age strata. Aggregated across severity, ConvNeXt+TCMH achieved accuracy/F1 of 0.961/0.874, 0.946/0.859, and 0.939/0.882 in the <60, 60–70, and >70 groups respectively, with the lowest uncertainty across all age groups (Supplementary Fig. S2C).

### Fast Progression Analysis

**Fig. 4.**
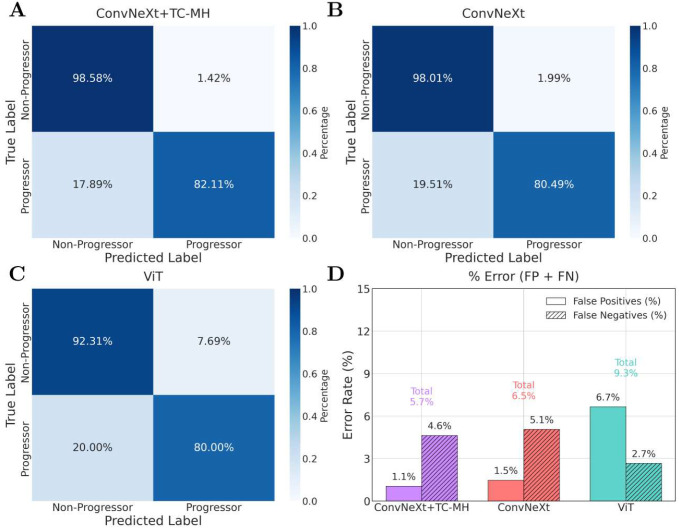
Fast progressor identification (MD slope ≤ −1 dB/year) across top-performing models. Normalized confusion matrices for (A) ConvNeXt+TCMH, (B) ConvNeXt, and (C) ViT on the fast progressor subset (116 eyes; 128 sequences). (D) Grouped bar chart comparing false positive and false negative error rates across the three models. ConvNeXt+TCMH achieves the lowest total error (5.7%), with error concentrated in false negatives (4.6%) rather than false positives (1.1%), whereas ViT shows a reversed error profile with substantially higher false positives (6.7%), reflecting reduced specificity in this high-risk subgroup.

Among the 971 progressor eyes, 116 (12%) met the fast progressor criteria (MD slope ≤ −1 dB/year), contributing 128 sequences to the analysis. In this high-risk subset, ConvNeXt+TCMH achieved the best overall performance (Fig. 4A), with specificity of 98.58% and sensitivity of 82.11%, yielding the lowest total error rate of 5.7% (false positive rate 1.1%; false negative rate 4.6%). ConvNeXt performed comparably (Fig. 4B; specificity 98.01%, sensitivity 80.49%, total error 6.5%; false positive rate 1.5%, false negative rate 5.1%). ViT showed a clinically distinct error profile characterised by a reversed error distribution, with false positives substantially exceeding false negatives (6.7% vs. 2.7%), alongside lower specificity (92.31%) despite comparable sensitivity (80.00%), resulting in the highest total error rate of 9.3% (Fig. 4C). Although differences in total error rates across models were not statistically significant (P > 0.05), ConvNeXt+TCMH consistently achieved the lowest error rate across both false positive and false negative components (Fig. 4D).

### Modality Attribution and Ablation Experiments

**Fig. 5.**
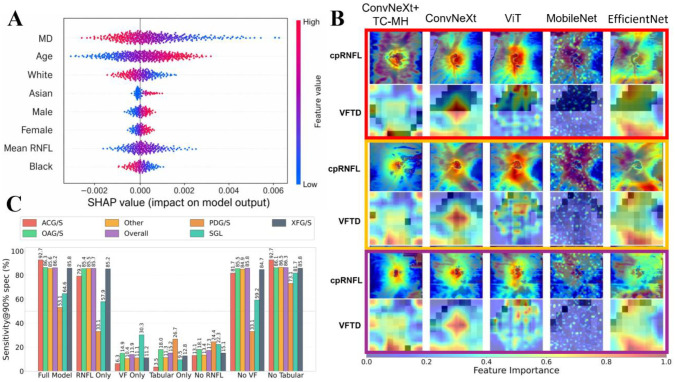
Feature attribution and ablation analysis for glaucoma progression prediction. (A) SHAP summary plot showing the distribution of feature effects on ConvNeXt+TCMH output across the cohort; each point represents one sample, positioned by its SHAP value and colored by the corresponding feature value. (B) Grad-CAM saliency maps averaged across 5-fold CV, shown for all five architectures grouped by demographic dimension: race (red box), sex (yellow box), and age (purple box), each comprising paired cpRNFL and VFTD activation rows. ConvNeXt+TCMH produced the most anatomically precise and demographically consistent activation, with focal optic nerve head saliency on cpRNFL maps and peripheral arcuate field weighting on VFTD maps. (C) Sensitivity at 90% specificity for ConvNeXt+TCMH across six glaucoma subtypes and the overall cohort, aggregated over 3-year prediction horizons and averaged across 5-fold CV. Multimodal gains are subtype-dependent, with particularly strong improvements for angle-closure and pigmentary glaucoma, and more limited gains for open-angle glaucoma. ACG/S = angle-closure glaucoma/suspect, OAG/S = open-angle glaucoma/suspect, PDG/S = pigmentary glaucoma/suspect, XFG/S = exfoliation glaucoma/suspect, and SGL = secondary glaucoma.

SHAP analysis of ConvNeXt+TCMH identified MD as the highest-ranked tabular predictor, with SHAP values spanning −0.002 to +0.006, the widest range among all features (Fig. 5A). High MD values were associated with strongly positive SHAP contributions (up to +0.006) and low values with negative contributions, indicating a monotonic relationship between functional severity and predicted progression risk. Age ranked second, followed by White race, Asian race, male sex, female sex, mean RNFL, and Black race in descending order of mean absolute SHAP importance. The SHAP value distributions for racial and sex features were narrow and centered near zero (range approximately ±0.001), and Black race had the lowest mean absolute SHAP value among all plotted features.

Grad-CAM saliency maps averaged across five folds are shown for all five architectures, grouped by demographic dimension: race (red box), sex (yellow box), and age (purple box), each comprising paired cpRNFL and VFTD activations (Fig. 5B). ConvNeXt+TCMH produced the highest peak activation (>0.8) with precise anatomical localization at the optic nerve head on cpRNFL maps and structured peripheral arcuate weighting on VFTD maps, both pathognomonic loci of glaucomatous neuroretinal loss. ConvNeXt showed comparable but slightly less focal localization (peak ~0.7–0.8). ViT generated diffuse, low-contrast activations (<0.5) without anatomical specificity; MobileNet produced coarse, spatially incoherent patterns; and EfficientNet exhibited sparse, low-magnitude saliency (<0.4) across both modalities. Critically, ConvNeXt+TCMH activation patterns were spatially invariant across race, sex, and age subgroups, indicating demographically robust and biologically grounded feature extraction.

Ablation analysis yielded an overall sensitivity at 90% specificity of 85.2% for the full multimodal model and 85.2% for the RNFL-only configuration (Fig. 5C). Sensitivity for the full model versus RNFL-only was 92.7% vs. 79.9% in ACG/S and 53.1% vs. 33.1% in PDG/S. Removing RNFL reduced sensitivity to 8.6–24.4% across subtypes, while removing VF yielded sensitivity of 76.4–85.8%. VF-only and tabular-only configurations achieved sensitivities of 30.3% and 15.1–26.7%, respectively.

### Model Calibration and Uncertainty Analysis

To assess prediction reliability for potential clinical translation, we performed comprehensive calibration and uncertainty analysis across all models.

**Fig. 6.**
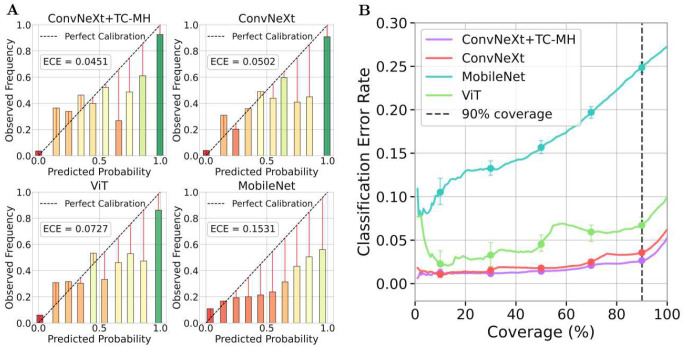
Calibration and uncertainty-aware selective prediction across all models. (A) Calibration curves showing observed frequency versus predicted probability for ConvNeXt+TCMH (ECE = 0.0451), ConvNeXt (ECE = 0.0502), ViT (ECE = 0.0727), and MobileNet (ECE = 0.1531). Red lines indicate calibration gaps between predicted and observed frequencies; dashed line denotes perfect calibration. (B) Risk-coverage curves quantifying classification error rate as a function of the fraction of retained predictions, ranked by Tsallis entropy uncertainty. ConvNeXt+TCMH achieves the lowest error across all coverage levels, followed closely by ConvNeXt, while MobileNet and ViT show substantially higher error rates, confirming that superior calibration translates directly to more reliable uncertainty-aware selective prediction. Vertical dashed line marks the 90% coverage clinical deployment threshold.

Calibration analysis (Fig. 6A) revealed that ConvNeXt+TCMH achieved the best calibration of all models (ECE = 0.0451; Table 3), with predicted probabilities closely aligned with observed frequencies across the full probability range. The ConvNeXt baseline showed comparable calibration (ECE = 0.0502), confirming that the TCMH training strategy preserves and marginally improves the calibration properties of the underlying backbone. ViT demonstrated acceptable but weaker calibration (ECE = 0.0727), while efficiency-optimized architectures exhibited substantially poorer calibration: MobileNet (ECE = 0.1531) showed moderate miscalibration and EfficientNet demonstrated severe miscalibration (ECE = 0.3043) with substantial prediction-accuracy gaps throughout the probability range.

Uncertainty quantification analysis further validated model reliability (Table 3). ConvNeXt+TCMH achieved the strongest uncertainty-accuracy separation, with the lowest accuracy drop from the lowest to highest uncertainty quartile (Q1 to Q4 drop = 0.132, *P* value < 0.001; Supplementary Fig. S3) and the highest uncertainty-error correlation (Pearson r = 0.369, *P* value < 0.001), confirming that Tsallis entropy reliably identifies unreliable predictions. The ConvNeXt baseline showed comparable but slightly weaker discrimination (accuracy drop = 0.137, r = 0.322, *P* value < 0.001), while ViT exhibited a larger accuracy drop across quartiles (0.196, P < 0.001) despite a lower correlation coefficient (r = 0.266), reflecting less consistent uncertainty estimation. MobileNet showed weaker uncertainty discrimination (accuracy drop = 0.333, r = 0.272), and EfficientNe’s severely compressed uncertainty range rendered confidence-based triage clinically uninformative for this architecture (accuracy drop = 0.334, r = 0.104). Demographic calibration disparity was lowest for ConvNeXt+TCMH (0.0163) and ConvNeXt (0.0166), substantially lower than ViT (0.0356), MobileNet (0.0275), and EfficientNet (0.1068), indicating lower subgroup calibration variability across patient subgroups (Table 2).

Risk-coverage analysis (Fig. 6B) demonstrated that ConvNeXt+TCMH achieved the lowest classification error rates across all coverage levels. At 50% coverage (retaining only the most confident predictions), ConvNeXt+TCMH maintained an error rate of 1.5%, compared with 2.0% for ConvNeXt, 2.5% for ViT, and 13.0% for MobileNet. At the clinically relevant 90% coverage threshold (vertical dashed line), ConvNeXt+TCMH achieved an error rate of 2.5%, followed by ConvNeXt (3.0%), ViT (4.5%), and MobileNet (6.5%). The ConvNeXt+TCMH model exhibited a gradual, well-controlled error increase as coverage approached 100% (final error rate 5.3%), whereas MobileNet showed substantially elevated error rates even under selective retention (10.0% at full coverage). The ConvNeXt baseline followed a nearly identical trajectory to ConvNeXt+TCMH (5.5% at 100% coverage), confirming that TCMH training does not compromise selective prediction performance while maintaining superior error control at clinically relevant operating points.

### Human-AI Comparative Validation Study

**Fig. 7.**
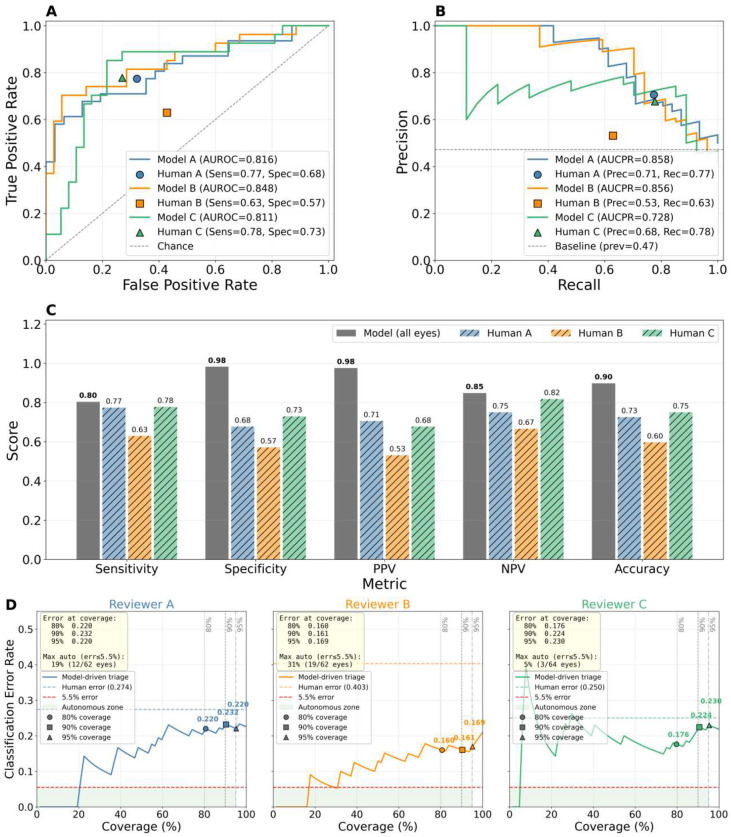
Model performance and clinical triage analysis against independent specialist reviewers. (A) ROC and (B) precision-recall curves for model predictions on each reviewer's test set; human grader operating points shown as markers with corresponding sensitivity/specificity and precision/recall. (C) Performance metrics against ground truth for the pooled model and each human grader; model achieves higher specificity and PPV while human graders show higher sensitivity. (D) Risk-coverage curves per reviewer showing model classification error as a function of retained coverage; markers indicate error rates at 80%, 90%, and 95% coverage thresholds; horizontal dashed lines show each reviewer's overall error rate; red dashed line marks the 5.5% autonomous classification threshold (De Fauw et al., *Nature Medicine*, 2018^15^); green shading indicates the autonomous zone where model error remains below this threshold. Reviewer A (blue), B (orange), C (green).

To assess real-world model applicability, we evaluated the model against three independent human graders on a held-out set of 108 unique eyes, comprising 805 longitudinal OCT-VF sequence pairs with accompanying tabular variables (RNFL thickness, mean deviation, age, sex, and race/ethnicity), none of which were used during model training or validation. Eyes were distributed across three graders, Reviewer A (62 eyes, 263 OCT-VF sequences), Reviewer B (62 eyes, 266 OCT-VF sequences), and Reviewer C (64 eyes, 276 OCT-VF sequences), with 30 eyes shared between each reviewer pair and 10 eyes common to all three graders to enable inter-rater reliability estimation. Representative longitudinal cpRNFLT and VF sequences for three agreement conditions (full model-grader concordance, full discordance, and partial agreement) drawn from this triple-overlap subset are shown in Supplementary Fig. S4, alongside per-visit MD and RNFLT values. For each sequence, graders were presented with longitudinal cpRNFLT maps and VF total deviation plots spanning up to five timepoints (T0-T4), alongside per-visit RNFLT, MD, and patient demographic information, and were asked to classify each eye as a progressor or non-progressor independently and without knowledge of the model output or ground truth label. Pairwise Cohen's kappa among graders ranged from 0.529 (Reviewer B vs. C, 76.7% agreement, n = 30) to 0.789 (Reviewer A vs. C, 90.0% agreement, n = 30), with Reviewer A vs. B yielding κ = 0.552 (80.0%, n = 30); Fleiss' kappa across all three graders on the 10-eye triple-overlap subset was 0.732, indicating substantial overall inter-rater reliability. On Reviewer A's 62-eye subset, the model achieved AUROC = 0.816 and AUCPR = 0.858 versus human sensitivity of 0.77 and specificity of 0.68, with model–human agreement of 67.7% (κ = 56.9%); on Reviewer B's 62-eye subset, the model attained the highest AUROC (0.848) and AUCPR (0.856) while the human grader reached sensitivity of 0.63 and specificity of 0.57, yielding model-human agreement of 56.5% (κ = 31.6%); on Reviewer C's 64-eye subset, the model achieved AUROC = 0.811 and AUCPR = 0.728, the human grader demonstrated sensitivity of 0.78 and specificity of 0.73, and this subset yielded the highest model-human agreement (80.7%, κ = 60.2%) as well as the highest human accuracy (0.953) and F1 score (0.945) across all reviewers. Aggregated across all eyes, the model achieved specificity of 0.98, PPV of 0.98, sensitivity of 0.80, NPV of 0.85, and accuracy of 0.90, exceeding all three human graders across every metric (Fig. 7C). Human vs. ground truth confusion matrices revealed that all three graders exhibited higher false-negative than false-positive rates, with worsening-eye sensitivity of 77.4% (Reviewer A), 63.0% (Reviewer B), and 77.8% (Reviewer C), and stable-eye misclassification rates of 32.3%, 42.9%, and 27.0%, respectively (Supplementary Fig. S4); in contrast, the model correctly classified 100% of stable eyes in the Reviewer A and B subsets and 94.6% in the Reviewer C subset, with worsening-eye sensitivity of 77.4%, 85.2%, and 96.3% across the three subsets. Under a model-driven triage framework adopting the 5.5% autonomous classification error threshold^15^, the model autonomously classified 19% (12/62), 31% (19/62), and 5% (3/64) of eyes across Reviewer A, B, and C subsets respectively without human intervention, and at 80% coverage achieved classification error rates of 0.220, 0.160, and 0.176, compared to human error rates of 0.274, 0.403, and 0.250, with errors remaining below 0.232 across all reviewers at 90% coverage (Fig. 7D, Table 4, Supplementary Fig. S5).

## Discussion

Our proposed ConvNeXt+TCMH framework advances glaucoma progression prediction in three scientifically important respects. First, it achieved strong discriminative performance among comparable prior methods summarized in Table 5, reaching an aggregated AUC of 96.8% in a cohort of 3,593 patients. Second, these gains were obtained in a substantially sparser and more clinically realistic setting, using only baseline and one follow-up visit to predict 2-, 3-, and 4-year progression, whereas most prior longitudinal methods required denser input histories. Third, our study extends beyond conventional discrimination metrics by jointly evaluating calibration, uncertainty stratification, demographic calibration disparity, and modality dependence, thereby positioning glaucoma progression prediction as a reliability-aware forecasting problem rather than solely a classification benchmark. Fourth, model performance was benchmarked against three independent specialist graders on 108 held-out eyes unseen during training, enabling direct quantification of model-human concordance, inter-rater reliability, and confidence-aware triage under realistic clinical conditions (Fig. 7, Table 4). Collectively, these distinctions indicate that the novelty of our framework lies not only in higher predictive performance, but in demonstrating that clinically interpretable long-horizon risk forecasting can be achieved from sparse multimodal observations under evaluation criteria more closely aligned with future clinical translation.

SHAP analysis identified MD and age as the two dominant tabular predictors, indicating that the tabular branch primarily captured baseline disease burden and age-associated risk rather than demographic covariates alone. The remaining features (White race, Asian race, male sex, female sex, mean RNFL, and Black race) contributed markedly smaller effects in descending order of importance, with Black race showing the lowest mean absolute SHAP value among all plotted features (Fig. 5A).

Grad-CAM saliency maps support biologically grounded feature extraction across all five architectures. ConvNeXt+TCMH produced the most anatomically precise activation, with focal optic nerve head saliency on cpRNFL maps and peripheral arcuate field weighting on VFTD maps (regions pathognomonic of glaucomatous structural and functional loss) that were spatially consistent across race, sex, and age subgroups, indicating the model learned anatomically meaningful representations rather than subgroup-specific shortcuts. ConvNeXt exhibited comparable but slightly less focal localization, while ViT, MobileNet, and EfficientNet produced progressively more diffuse, coarse, or heterogeneous activations (Fig. 5B). The superior spatial precision of ConvNeXt+TCMH is likely attributable to the TCMH loss, which enforces monotonically ordered risk across the 2-, 3-, and 4-year prediction horizons. By requiring internally consistent probability trajectories, this constraint functions as a biologically grounded regularizer that penalizes feature representations incompatible with the irreversible, progressive nature of glaucomatous damage, selectively reinforcing structural features at clinically relevant anatomical loci while suppressing spurious activations. This temporal consistency objective may therefore drive the encoder toward representations that better capture disease heterogeneity across subtypes and demographic groups, explaining both the improved saliency precision and the subgroup-invariant activation patterns observed relative to single-horizon baselines.

At the modality level, structural imaging carried the dominant predictive signal, with RNFL-only performance nearly matching the full multimodal model overall, while RNFL removal caused catastrophic degradation across all subtypes. Removing VF preserved substantially higher performance, confirming it as a complementary but secondary modality for long-horizon prediction. These findings support the interpretation that future glaucoma worsening is predominantly encoded in structural RNFL signatures detectable from early visits, with functional measurements refining rather than driving the prognostic signal. Multimodal benefit was strongly subtype-dependent rather than uniform. RNFL alone sufficed for OAG/S and XFG/S, suggesting that in these common subtypes, structural damage patterns are sufficiently informative to predict future worsening without additional functional or clinical input. In contrast, multimodal fusion provided the largest gains in PDG/S (a subtype characterized by distinct pathophysiology, atypical optic nerve morphology, and less tightly coupled structure–function trajectories) where sensitivity increased by 20.0 percentage points over RNFL alone. This pattern suggests that structural RNFL measurements are less independently sufficient when disease mechanisms diverge from the canonical open-angle damage model. These findings argue against a universal multimodal strategy and instead support a subtype-aware framework in which the incremental value of each modality is weighted according to the underlying disease biology (Fig. 5C).

Among the evaluated backbones, ConvNeXt+TCMH provided the most favorable balance between discrimination and predictive reliability. Although the gain in raw classification performance over the ConvNeXt baseline was modest, TCMH yielded consistent improvements in probability quality, uncertainty stratification, and subgroup robustness. ConvNeXt+TCMH achieved the lowest expected calibration error (ECE = 0.0451) and the strongest uncertainty–error coupling (r = 0.3685) among all evaluated models, while maintaining the smallest maximum demographic calibration disparity (Table 3 and Fig. 6A). These findings suggest that imposing temporally ordered risk across prediction horizons acts as a biologically grounded regularizer, encouraging outputs that are better calibrated and more clinically interpretable rather than simply more discriminative. This reliability profile has direct implications for clinical deployment, where decisions depend not only on rank ordering of risk but on whether high-confidence predictions can be acted on safely and low-confidence predictions appropriately escalated for expert review. The risk-coverage analysis at the 90% coverage threshold (Fig. 6B, vertical dashed line) illustrates this trade-off in a clinically relevant operating scenario: ConvNeXt+TCMH maintained an error rate of only 2.5% while covering 90% of the population, compared with 3.0% for ConvNeXt, 4.5% for ViT, and 6.5% for MobileNet. This indicates that in a deployment setting where 10% of cases with the highest uncertainty are flagged for expert review, ConvNeXt+TCMH would achieve a substantially lower error rate on automatically processed predictions than alternative architectures, supporting more reliable confidence-aware triage. By contrast, EfficientNet exhibited marked miscalibration despite being a contemporary architecture, illustrating that modern model design does not guarantee clinically meaningful probability estimates. Taken together, these results indicate that architecture selection for glaucoma progression forecasting should be guided by calibration quality and uncertainty behavior in addition to conventional discrimination metrics, particularly when models are intended for confidence-aware triage or treatment planning.

Benchmarking against three independent specialist graders on 108 held-out eyes with 805 longitudinal OCT-VF sequence pairs with accompanying tabular variables (RNFL thickness, mean deviation, age, sex, and race/ethnicity) revealed that inter-rater agreement was itself substantial but imperfect (Fleiss' κ = 0.732), confirming that longitudinal multimodal progression classification carries inherent ambiguity even among experienced clinicians. The model consistently exceeded human graders on specificity and PPV across all reviewer subsets, while human graders showed competitive sensitivity at the cost of markedly higher false-positive rates, a systematic over-calling bias under uncertainty that the model does not share. This complementary error structure suggests that the greatest clinical utility of ConvNeXt+TCMH lies not in replacing specialist judgment but in a confidence-stratified triage architecture: autonomously resolving high-confidence stable cases while escalating uncertain worsening predictions for expert review. The risk-coverage analysis (Fig. 7D) operationalizes this directly, demonstrating that model error rates remained below human error rates at all coverage thresholds and that a meaningful case fraction could be autonomously resolved under the established 5.5% error constraint^15^, with residual variation across reviewer subsets attributable to case difficulty distribution rather than model instability (Table 4).

Several limitations should be considered when interpreting these findings. First, progression labels were defined using horizon-specific longitudinal outcomes, such that the model predicts future progression status from multi-horizon visits rather than classifying against a single static endpoint. Although this design closely reflects clinical reality, it also means that label quality depends on the stability and reproducibility of longitudinal VF-based progression assessment. Second, the fast-progressor subgroup was comparatively small, which limits statistical precision for subgroup-level inferences in that setting. Third, the sliding-window design generated multiple sequences per eye; although patient-level cross-validation prevents leakage across folds, residual within-eye correlation may modestly inflate performance relative to a fully independent prospective design. Finally, external validation on an independent multimodal longitudinal cohort was not feasible at the time of this study, as no existing public repository, including large biobanks such as UKBB, provides the combination of longitudinal cpRNFL OCT, VF total deviation maps, and multi-year follow-up required by the sparse-observation framework, reflecting a recognized gap in the field rather than a study-specific constraint. Future work should therefore focus on prospective multicenter data collection, integration of additional structural biomarkers such as macular OCT, and development of time-aware encoders that more explicitly model irregular follow-up intervals while preserving the calibration and uncertainty advantages observed here.

## Conclusion

In this retrospective study, we demonstrate that reliable glaucoma progression forecasting across 2-, 3-, and 4-year horizons is achievable from only two clinical visits by integrating structural OCT, functional visual field data, and clinical covariates within a sparse-observation multimodal framework, the minimum observation requirement among comparable longitudinal approaches. ConvNeXt+TCMH delivered the strongest joint performance across discrimination, calibration, uncertainty stratification, and demographic consistency, with the temporally consistent multi-horizon objective functioning as a biologically grounded regularizer that improved probability reliability without sacrificing predictive accuracy. Although RNFL carried the dominant prognostic signal overall, multimodal fusion provided subtype-dependent gains concentrated in angle-closure and pigmentary glaucoma, arguing against a universal imaging strategy in favor of disease-biology-aware modality weighting. Benchmarked against three independent specialist graders, the model exceeded human performance on specificity and PPV while its calibrated uncertainty estimates supported a viable confidence-stratified triage architecture. Taken together, these findings position multi-horizon glaucoma risk stratification as a clinically tractable problem, while underscoring that responsible deployment will require prospective multicenter validation and subtype-aware uncertainty-informed decision frameworks.

## Materials and Methods

This study was approved by the Mass General Brigham Institutional Review Board and adhered to the Declaration of Helsinki; informed consent was waived given the retrospective design.

### Study Population and Data Preparation

We identified glaucoma patients seen at Massachusetts Eye and Ear between 2010 and 2023 (26,562 patients with 217,041 VFs; 46,963 patients with 320,221 CpRNFL OCT scans). CpRNFL thickness was measured using the Zeiss Cirrus HD-OCT (Carl Zeiss Meditec, Dublin, CA) and VF testing performed with the Humphrey Field Analyzer II SITA 24–2 protocol^32^. Eyes were classified as glaucomatous using PyGlaucoMetrics^33^ (probability ≥0.5, ≥2 glaucomatous VFs); suspects and preperimetric cases were excluded. Glaucoma subtypes (OAG/S, ACG/S, XFG/S, PDG/S, SGL) were determined using a validated large language model applied to clinical notes^34^.

Eligible eyes required at least two reliable 24–2 VF tests (false-positive rate <33%) and corresponding OCT scans (signal strength ≥7) within the first year after baseline, with ≥3 years of total follow-up. VF and OCT records were matched by patient ID, laterality, and exam date within ±6 months. Baseline covariates (age, sex, race, MD, average CpRNFL) were extracted from structured metadata, yielding an intermediate cohort of 7,943 patients (11,338 eyes; 48,664 VF-CpRNFL pairs). Applying sequence generation requirements with complete paired data at T_0_ and T_1_ (±0.25-year tolerance) and at least one valid prediction visit (±0.75-year tolerance) yielding the final analytic dataset of 5,645 eyes from 3,593 patients contributing 13,087 sequences. Progression labels were assigned using the majority ensemble criterion described in the following section. Class imbalance was addressed through weighted loss during training. VFTD values were used as model input. When not directly available, TD values were computed from pointwise sensitivity measurements using PyVisualFields^33^ and spatially mapped onto an 8×9 grid corresponding to the HFA 24–2 layout, with blind spot locations masked (Supplementary Fig. S6). All VF and CpRNFL images were resized to 224×224 pixels and normalized to ImageNet statistics for compatibility with pretrained backbones. Tabular covariates were preprocessed separately: categorical variables were one-hot encoded and continuous features z-score normalized using fold-specific scalers to prevent leakage. Missing visits within the observation window were handled by nearest-neighbor time matching within ±6 months.

### Progression Label Assignment and Sequence Construction

Two progression label levels were defined: 1) eye-level labels from all available longitudinal VF data, and 2) sequence-level horizon-specific labels assigning progression status at years 2, 3, and 4.

**Fig. 8.**
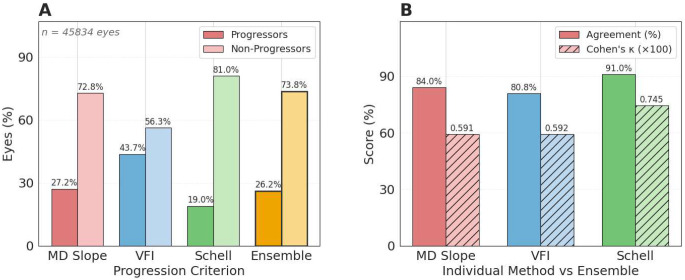
Progression label derivation using majority ensemble of three criteria. (A) Proportion of eyes labeled as progressors and non-progressors by each individual method (MD slope^37^, VFI^38^, Schell^39^) and the majority ensemble (≥2 of 3), showing method-specific variation in progression rates that motivates ensemble labeling. (B) Agreement rate (%) and Cohen's κ between each individual method and the majority vote label, confirming high consensus and label robustness across criteria. MD = mean deviation; VFI = visual field index.

**Fig. 9.**
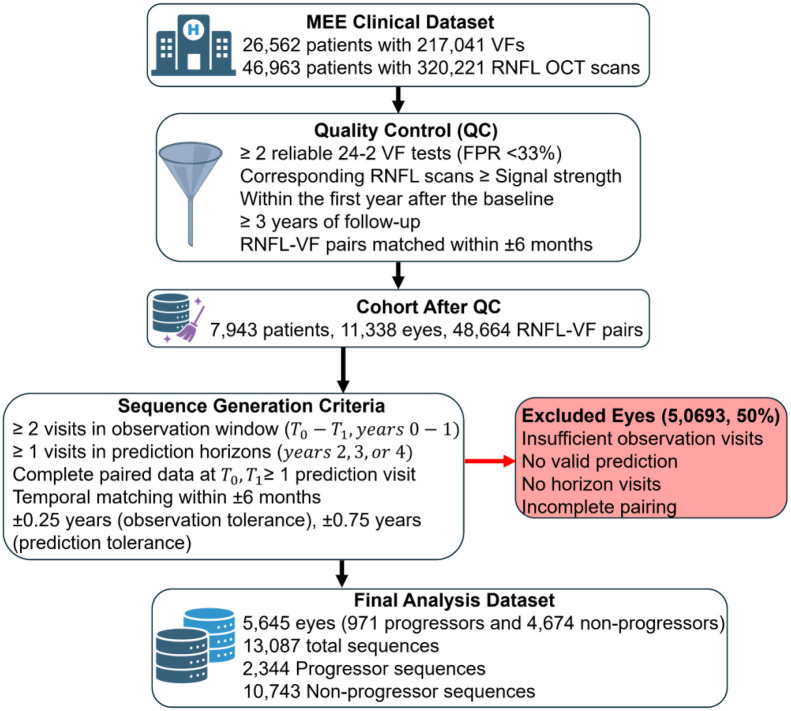
Flowchart of data curation, cohort selection, and sequence generation.

A sliding window generates multiple four-year sequences per eye, each comprising a one-year observation window (inputs) followed by a three-year prediction interval^29^. Each sequence uses exactly two visits as model input (baseline (T_0_) and one follow-up (T_1,_ ~1 year later)) identified by nearest-neighbor time matching. Prediction labels were assigned at years 2, 3, and 4 using the closest visit within ±0.75-year tolerance; if no valid visit existed for a given horizon, no label was assigned rather than excluding the sequence. Progression labels were derived using the vfprogression package^35,36^ by combining three validated criteria: MD slope^37^, VF Index (VFI)^38^, and the Schell^39^ criterion, each applied to measurements accumulated from T0 to each prediction horizon. An eye was labeled as progressing if any two of the three criteria indicated progression (majority ensemble). This ensemble approach was adopted to improve label robustness over any single criterion, as each method captures distinct aspects of glaucomatous functional decline. The agreement across individual progression criteria and the majority vote is shown in Fig. 8. Applying these ensemble labels to the 13,087 sequences yielded 971 progressor eyes and 4,674 non-progressor eyes (4.8:1 non-progressor/progressor ratio). The complete cohort selection and label assignment pipeline is summarized in Fig. 9. Applying these ensemble labels to the 13,087 sequences yielded 971 progressor eyes (2,713 sequences) and 4,674 non-progressor eyes (10,374 sequences), corresponding to the 4.8:1 class imbalance noted above.

Using these labeled visits, each sequence was formally defined as follows. The multimodal input X_T0_,T_1_ comprised paired structural and functional observations at both visits:

(1) $$X_{T_{0},T_{1}}=\left\{\left(X_{T_{0}}^{\text{RNFL}},X_{T_{0}}^{VF},X_{T_{0}}^{tab}\right),\left(X_{T_{1}}^{\text{RNFL}},X_{T_{1}}^{VF},X_{T_{1}}^{tab}\right)\right\}$$

with the corresponding horizon-specific label vector:

(2) $$y=\left(y_{2},y_{3},y_{4}\right)$$

where each y_*h*_ ∈ {0,1}denotes progression status at year *h* ∈ {2,3,4}.

**Fig. 10.**
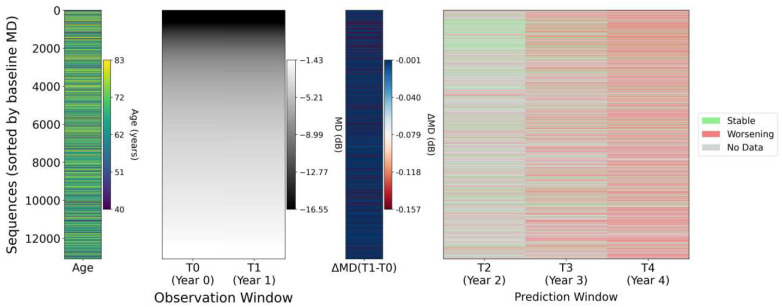
Extracted sequences and horizon-specific labels. VF MD values during observation (left) and corresponding horizon-specific progression labels (right).

At eye level, progressors were defined as eyes meeting the majority ensemble criterion (≥2 of 3: MD slope, VFI, Schell) for statistically significant VF worsening; a fast progressor subset was defined as MD slope ≤ −1.0 dB/year^40^, calculated using linear mixed-effects models with covariates including age, baseline severity, sex, race, and follow-up duration. Training and evaluation use sequence-level labels; eye-level labels are used only for statistical reporting. Accordingly, all primary model metrics reported below are sequence-level unless otherwise noted. Fig. 10 visualizes the extracted sequences and their horizon-specific labels.

### Model Architecture and Training Protocol

The framework jointly processes cpRNFL images, VFTD images, and tabular covariates from two visits (T_0_ and T_1_). At each visit, cpRNFL and VF images are encoded using a shared-weight ConvNeXt backbone, and the resulting image embeddings are concatenated with a tabular embedding generated by a two-layer multilayer perceptron applied to age, sex, race, baseline MD, and mean RNFL. These visit-level multimodal representations are then passed to a two-layer bidirectional LSTM with 256 hidden units per direction and dropout of 0.3. The final temporal representation is combined with learned horizon embeddings and passed through a shared prediction head followed by a linear output layer to generate three sigmoid probabilities corresponding to progression at years 2, 3, and 4:

(3) $$\hat{\text{y}}=\left[{\hat{\text{y}}}_{2},{\hat{\text{y}}}_{3},{\hat{\text{y}}}_{4}\right]=Net\left(X_{T_{0},T_{1}}\right)$$

Accordingly, the probability of non-progression at horizon h is given by $1-{\hat{\text{y}}}_{h}$. Unlike a standard multi-output classifier, the proposed model incorporates horizon-specific embeddings and a temporally consistent multi-horizon loss, allowing each prediction to remain horizon-aware while respecting the expected monotonic increase in progression risk over time.

For the proposed model, the ConvNeXt backbone was implemented as ConvNeXt base with pretrained weights, while the same sparse-observation multi-horizon framework was also evaluated using alternative visual encoders in separate experiments for comparative benchmarking. Training used AdamW with a learning rate of 2 × 10^−5^, weight decay of 1 × 10^−2^, batch size 16, linear warm-up for 10 epochs, and a maximum of 200 epochs with early stopping patience of 11 epochs. The loss function combined class-weighted binary cross-entropy across the three prediction horizons with a temporal monotonicity penalty:

(4) $$L_{\text{total}}=L_{BCE}+\lambda_{\text{mono}}L_{\text{mono}}$$

where *λ*_*mono*_ = 0.5 (selected to ensure that the temporal consistency constraint meaningfully regularized predictions without dominating the binary classification objective) and *L*_*mono*_ penalizes violations of ${\hat{\text{y}}}_{2}\leq {\hat{\text{y}}}_{3}\leq {\hat{\text{y}}}_{4}$. Positive-class weights were computed separately for each horizon from the training fold to address class imbalance. Because the final cohort construction required valid labels at all three horizons, no missing-label masking was applied during TCMH training in this implementation. Full model configurations and hyperparameters are shown in Supplementary Table S1.

Strict patient-level five-fold cross-validation was used throughout to prevent data leakage across folds. Cohort construction required two observation visits within the allowed observation window and valid progression labels at years 2, 3, and 4 within the specified prediction tolerance, ensuring that the TCMH model was trained and evaluated on the same sequence definition as the baseline architectures. Fold assignments were fixed across experiments to enable fair model comparison. Continuous tabular covariates were standardized within each training fold using a StandardScaler fit only on training visits, and the fitted scaler was then applied to validation data. Data augmentation during training included random horizontal flips and rotations up to 10 degrees, whereas validation images were resized and normalized without augmentation. The training procedure including the pseudocode is illustrated in Supplementary Fig. S7A. Training loss converged consistently across all five folds, with both total loss and the monotonicity penalty (L_mono_) stabilizing within 50 epochs, confirming training stability and the absence of fold-specific divergence (Supplementary Fig. S7B)

### Statistical Analysis and Performance Metrics

Model discrimination was evaluated primarily by the area under the receiver operating characteristic curve (AUROC), computed separately for each prediction horizon and aggregated across the five patient-level cross-validation folds. Secondary classification metrics included accuracy, F1-score, and area under the precision-recall curve (AUPRC). For selected subgroup and fast-progressor analyses, sensitivity, specificity, false-positive rate, false-negative rate, and total error were additionally reported where clinically relevant. In the modality ablation experiments, performance was summarized as sensitivity at 90% specificity to reflect a clinically conservative operating point. Unless otherwise stated, results are reported as mean ± SD across folds.

Calibration and uncertainty were evaluated to assess clinical reliability beyond discrimination alone. Calibration was quantified using expected calibration error (ECE), and demographic reliability was summarized using the maximum subgroup calibration disparity across race, sex, and age strata. Prediction uncertainty was quantified using Tsallis entropy with q = 0.25, which increases sensitivity to low-probability variations and improves discrimination of uncertainty in imbalanced classification settings. Lower values of q emphasize deviations in small, predicted probabilities, making the measure particularly responsive to uncertainty in minority-class predictions such as progression events. This choice is consistent with prior work demonstrating that Tsallis entropy with q < 1 enhances sensitivity to rare-event uncertainty and provides more informative uncertainty estimates than Shannon entropy in skewed distributions^30,31^. For binary prediction with class probabilities pi, uncertainty was computed as:

(5) $$\left(\frac{1}{q-1}\right)\times (1-\sum_{i=0}^{n=1}log({P_{i}}^{q})$$

Risk-coverage analysis was used to evaluate selective prediction behavior by measuring classification error as a function of retained sample fraction after excluding the most uncertain predictions. Uncertainty quality was further assessed by the accuracy drop from the lowest-uncertainty quartile (Q1) to the highest-uncertainty quartile (Q4), and by the Pearson correlation between uncertainty and prediction error.

Feature attribution for tabular predictors was assessed using SHAP. Results were summarized using both per-sample SHAP distributions and mean absolute SHAP values to quantify the relative contribution of baseline MD, age, race, sex, and mean RNFL to model output. Modality contribution was further evaluated through systematic ablation of RNFL, VF, and tabular inputs across subtypes and the overall cohort.

All statistical tests were two-sided, with *P* < 0.05 considered statistically significant. Architecture-level comparisons across folds were performed using paired t-tests. Subgroup analyses across race, sex, and age categories used analysis of variance (ANOVA) followed by Bonferroni-corrected post hoc t-tests where appropriate. Correlations between uncertainty and error were assessed using Pearson correlation coefficients. All analyses were conducted in Python 3.10 using SciPy v1.13 and statsmodels v0.14.

## Supplementary Material

Supplementary Files

This is a list of supplementary files associated with this preprint. Click to download.

- SupplementNCfinalver.docx

## Figures and Tables

**Fig. 2.**
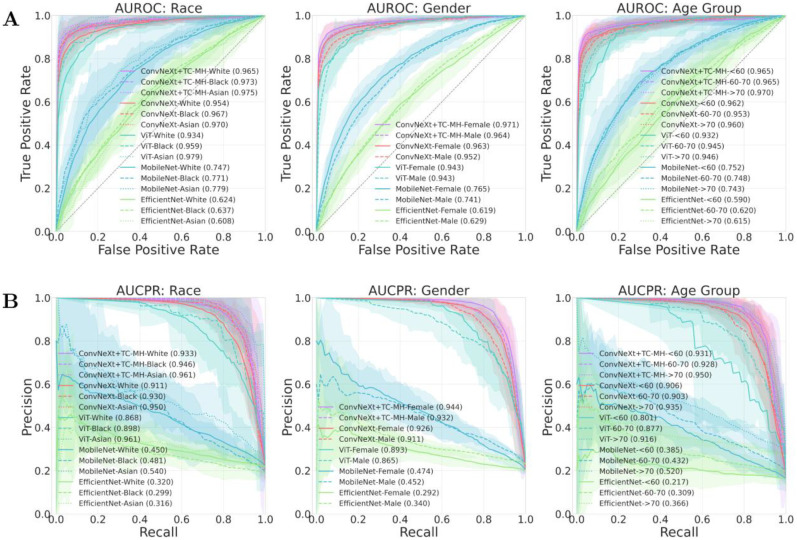
Subgroup performance across demographics. AUROC (A) and AUCPR (B) curves stratified by race, gender, and age group for all models. ConvNeXt+TCMH consistently achieves the highest discrimination and precision across subgroups, with reduced variability compared to baseline architectures.

**Table 1.** Baseline demographics and clinical characteristics (Mean ± SD or n (%); t-test/chi-square; *p* < 0.05).

Characteristic	Progressors (971 eyes)	Non-progressors (4,674 eyes)	*P*-value
Age at baseline, years	70.5 ± 12.8	65.6 ± 14.7	<0.001
Gender, n(%)			
Female	496(51.1)	2,711(58)	
Male	475(48.9)	1,963(42)	
Race, n(%)			<0.001
Asian	74(7.6)	351(7.5)	
Black/African American	144(14.8)	785(16.8)	
White	676(69.6)	2,968(63.5)	
Other	77(8)	570(12.2)	
Follow-up time, years	2.1 ± 1	2.1 ± 1	ns*
Mean TD at baseline, dB	−7.82 ± 5.98	−5.78 ± 5.08	<0.001
Mean MD at baseline, dB	−8.11 ± 6.24	−5.9 ±5.25	<0.001
MD slope at baseline, dB/year	−0.013	−0.005	<0.001
Mean CpRNFL at baseline, μm	59.4 ± 9.5	77.9 ± 11.3	<0.001
CpRNFL slope at baseline, μm/year	−0.530	−0.264	<0.001

* ns = not significant.

**Table 2.** Severity-stratified performance and uncertainty across models. Values are averaged across race, sex, and age subgroups within each severity stage. ConvNeXt+TCMH consistently achieves the highest discrimination and F1-score with the lowest uncertainty across all stages, indicating improved robustness and reliability relative to baseline architectures. Bold values indicate the best performance.

Severity	Model	AUROC	AUPRC	Accuracy	F1-score	Uncertainty
**Stage 1**	ConvNeXt+TCMH	**0.950**	**0.879**	**0.963**	**0.824**	**0.195**
	ConvNeXt	0.930	0.838	0.955	0.791	0.265
	ViT	0.926	0.812	0.928	0.769	0.381
	MobileNet	0.678	0.238	0.770	0.296	0.704
	EfficientNet	0.526	0.129	0.279	0.194	0.909
**Stage 2**	ConvNeXt+TCMH	**0.958**	**0.923**	**0.949**	**0.876**	**0.193**
	ConvNeXt	0.950	0.908	0.940	0.851	0.265
	ViT	0.904	0.837	0.901	0.778	0.394
	MobileNet	0.706	0.394	0.739	0.443	0.711
	EfficientNet	0.526	0.235	0.331	0.333	0.908
**Stage 3**	ConvNeXt+TCMH	**0.965**	**0.955**	**0.934**	**0.898**	**0.191**
	ConvNeXt	0.904	0.942	0.923	0.881	0.256
	ViT	0.904	0.853	0.880	0.800	0.394
	MobileNet	0.691	0.536	0.673	0.508	0.720
	EfficientNet	0.555	0.388	0.406	0.472	0.908

**Table 3.** Calibration, uncertainty quantification, and demographic disparity metrics across all models. Bold values indicate the lowest (best) value per metric column.

Model	ECE	Accuracy drop (Q1 to Q4)	Uncertainty correlation	Demographic disparity (Max)
**ConvNeXt+TCMH**	**0.0451**	**0.1321**	**0.3685**	**0.0163**
ConvNeXt^22^	0.0502	0.1368	0.3224	0.0166
ViT^23^	0.0727	0.1960	0.2664	0.0356
MobileNet^24^	0.1531	0.3328	0.2715	0.0275
EfficientNet^25^	0.3043	0.3343	0.1038	0.1068

**Table 4.** Performance comparison between the proposed model and independent specialist graders across reviewer-specific held-out test sets.

	N of eyes (sequences)	Accuracy	F1 Score	AUROC	AUCPR	Kappa (%)	Agreement (%)
**RA**	62 (263)	0.887	0.873	0.816	0.858	56.864	67.742
**RB**	62 (266)	0.935	0.920	**0.848**	**0.856**	31.622	56.452
**RC**	64 (276)	**0.953**	**0.945**	0.811	0.728	**60.216**	**80.688**

RA=Reviewer A; RB=Reviewer B; RC=reviewer C. Bold values indicate the best value per metric column.

**Table 5.** Benchmarking against state-of-the-art glaucoma progression prediction methods. Bold values show the best performance.

Author, year	Dataset	Model	Observation Window (visits)	Prediction window (years)	Mean Accuracy (%)	Mean AUC (%)
Yousefi et al., 2014^19^	180 eyes (107 progressing, 73 stable), longitudinal OCT CpRNFL + VF data	Random Forest, Bayes Net, CART, etc.	Multiple visits, tabular features	2	N/A	88
Dixit et al., 2021^9^	11,242 eyes (longitudinal VF + clinical)	Convolutional LSTM	≥4 consecutive VF visits	4	93	89–93
Tarcoveanu et al., 2022^20^	50 patients (100 eyes, 3 visits) + 101 eyes (with diabetes)	Random Forest, MLP, kNN, SVM, etc.	3 visits (baseline + 2 follow-ups)	3	92	N/A*
Afolabi et al., 2025^21^	500 patients with 100,000 OCT B-scans	FairDist with EfficientNet backbone	1 visit (baseline OCT only)	6	N/A	74
Chen et al., 2025^10^	3,871 patients (28,943 VFs from 7,248 eyes)	VF-Transformer with ICDT loss	≥5 longitudinal VF visits	N/A	90.7	92.6
**This study, 2026**	3,593 patients (5,645 eligible eyes) with 13,087 sequences (48,664 CpRNFL-VF pairs)	Sparse-Observation with temporally consistent multi-horizon loss	2 visits (baseline + 1 follow-up) ★	2–4	**94.7**	**96.8**

★ Minimum observation requirement among all multimodal longitudinal frameworks.

* N/A= Not applicable.

## Data Availability

The data that support the findings of this study can be obtained from the corresponding author upon reasonable request.

## Code Availability

The codes used in this study are publicly available in the first author’s GitHub page at: https://github.com/Mousamoradi/Sparse-Observation-Multi-Horizon-Progression.
